# Tear Lipocalin and Lipocalin-Interacting Membrane Receptor

**DOI:** 10.3389/fphys.2021.684211

**Published:** 2021-08-19

**Authors:** Ben J. Glasgow

**Affiliations:** Departments of Ophthalmology, Pathology and Laboratory Medicine, Jules Stein Eye Institute, University of California, Los Angeles, Los Angeles, CA, United States

**Keywords:** tear lipocalin, lipocalin interacting membrane receptor, lipocalin 1, von Ebner's gland protein, gene sharing, human tears, limb development membrane protein-1, phospholipid transfer protein

## Abstract

Tear lipocalin is a primate protein that was recognized as a lipocalin from the homology of the primary sequence. The protein is most concentrated in tears and produced by lacrimal glands. Tear lipocalin is also produced in the tongue, pituitary, prostate, and the tracheobronchial tree. Tear lipocalin has been assigned a multitude of functions. The functions of tear lipocalin are inexorably linked to structural characteristics that are often shared by the lipocalin family. These characteristics result in the binding and or transport of a wide range of small hydrophobic molecules. The cavity of tear lipocalin is formed by eight strands (A–H) that are arranged in a β-barrel and are joined by loops between the β-strands. Recently, studies of the solution structure of tear lipocalin have unveiled new structural features such as cation-π interactions, which are extant throughout the lipocalin family. Lipocalin has many unique features that affect ligand specificity. These include a capacious and a flexible cavity with mobile and short overhanging loops. Specific features that confer promiscuity for ligand binding in tear lipocalin will be analyzed. The functions of tear lipocalin include the following: antimicrobial activities, scavenger of toxic and tear disruptive compounds, endonuclease activity, and inhibition of cysteine proteases. In addition, tear lipocalin binds and may modulate lipids in the tears. Such actions support roles as an acceptor for phospholipid transfer protein, heteropolymer formation to alter viscosity, and tear surface interactions. The promiscuous lipid-binding properties of tear lipocalin have created opportunities for its use as a drug carrier. Mutant analogs have been created to bind other molecules such as vascular endothelial growth factor for medicinal use. Tear lipocalin has been touted as a useful biomarker for several diseases including breast cancer, chronic obstructive pulmonary disease, diabetic retinopathy, and keratoconus. The functional possibilities of tear lipocalin dramatically expanded when a putative receptor, lipocalin-interacting membrane receptor was identified. However, opposing studies claim that lipocalin-interacting membrane receptor is not specific for lipocalin. A recent study even suggests a different function for the membrane protein. This controversy will be reviewed in light of gene expression data, which suggest that tear lipocalin has a different tissue distribution than the putative receptor. But the data show lipocalin-interacting membrane receptor is expressed on ocular surface epithelium and that a receptor function here would be rational.

## Introduction

**Figure 1:**
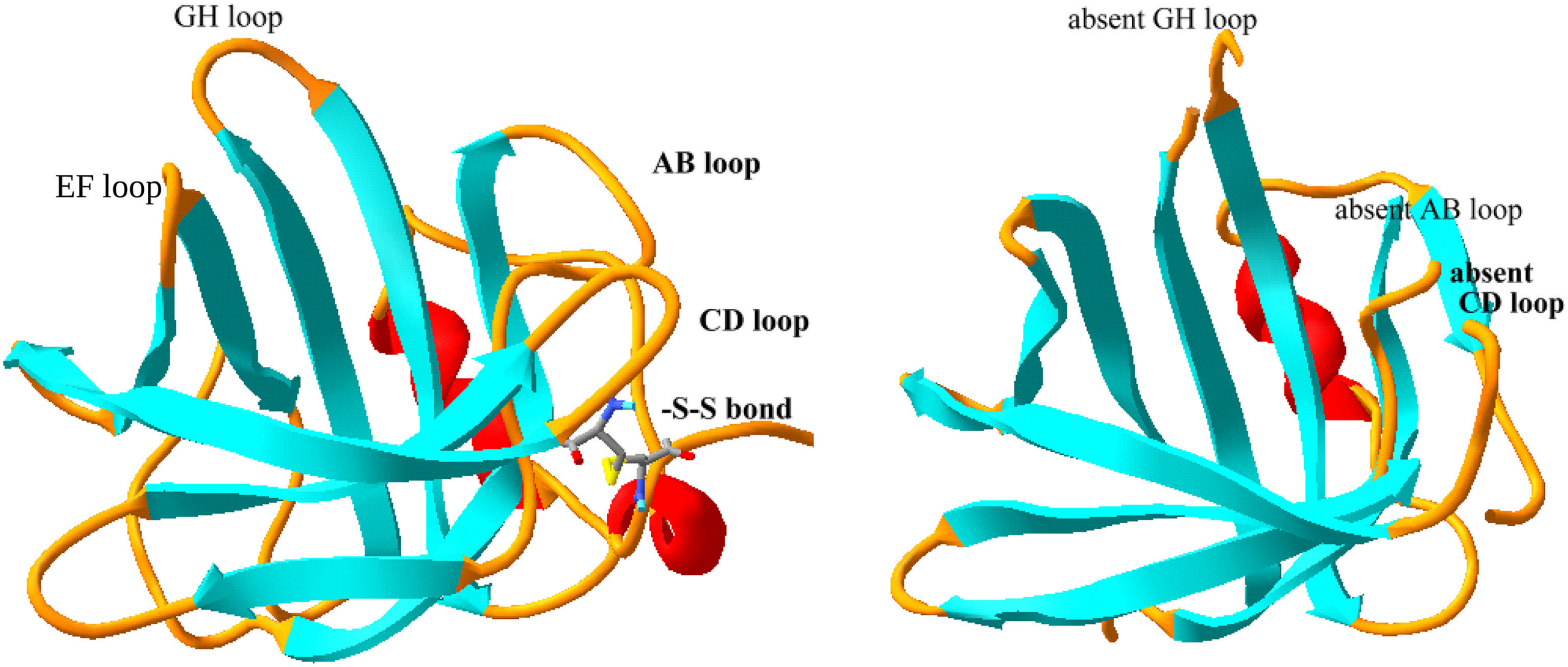
Comparison of solution and crystal structure of tear lipocalin. The solution structure is shown on the left (Gasymov et al., 2001) and the crystal structure is shown on the right (Breustedt et al., 2005), PDB file, 1XKI. The loops (yellow) were evident from the solution structure, but were not resolved by the crystal structure. The strands or β sheets are shown in aqua and the α-helices are shown in red. The loops are named (black) by the adjoining strands for orientation.

**Figure 2:**
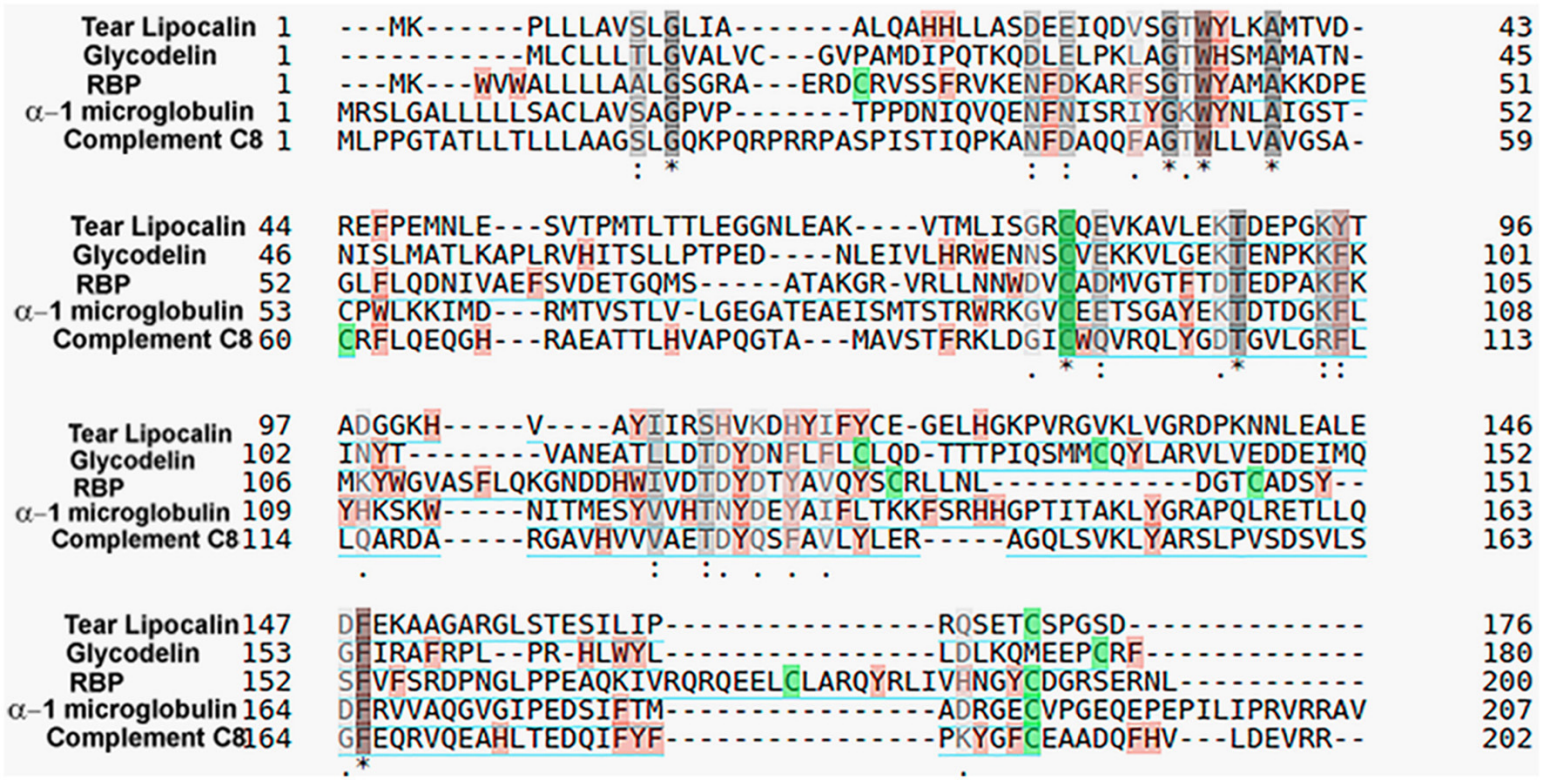
Comparison of representative human lipocalins for amino acid sequence identity. Colors indicate disulfide bonds (green), aromatic residues (tan), and similar amino acid properties (gray). Asterisk (^*^) indicates a fully conserved sequence, colon (:) indicates groups of strongly similar properties and period (.) indicates groups of weakly similar properties. Sequence identity was determined by the Universal Protein Resource (https://www.uniprot.org/align/) with protein accession numbers: P31025-tear lipocalin, P09466-glycodelin, P02753-retinol-binding protein 4 (RBP), and P07360-complement component C8 gamma chain.

Tear lipocalin is a member of the calycin superfamily, which includes fatty acid binding proteins, avidins, and the lipocalin family. The lipocalins share a highly conserved lipocalin fold formed by eight antiparallel β strands that are continuously hydrogen-bonded and folded in the shape of a flattened pita bread (Flower, 1996). These strands in concert with conserved 3_10_ and α-helices form the internal ligand-binding site for small hydrophobic ligands. Lipocalins are small; the main isoform of tear lipocalin has a molecular mass 17,446 Da (Glasgow et al., 1998b). The published solution and crystal structures of tear lipocalin are shown in Figure 1 (Gasymov et al., 2001; Breustedt et al., 2005). As with other lipocalins, tear lipocalin contains the highly conserved regions of the lipocalin family, including the 3_10_ helix (preceding the A strand), the FG loop, and the α-helix preceding the I strand. The structures show a capacious and flexible pita-shaped cavity. Hydrophobic residues line the internal cavity including a highly conserved tryptophan (Figure 2). Tear lipocalin is a multifaceted protein with a variety of functions. At least four prior reviews updated its functions. The most recent was 10 years ago (Redl, 2000; Glasgow et al., 2002c; Dartt, 2011; Glasgow and Gasymov, 2011). As noted in one of the reviews, the functions in tears need to be rectified with the potential receptor on the ocular surface (Dartt, 2011). Recent advances have been made in our understanding of the structure and functions of tear lipocalin as well as its putative receptor. This review will update the prior reviews with a focus on new information, including some unpublished data. The methods and discussion section will present some new data regarding the lipocalin-interacting membrane receptor.

### Nomenclature

Tear lipocalin was originally discovered and named according to its electrophoresis profiles of tears. Initially referred to as “tear albumin” (Erickson et al., 1956), later the name was changed to “anodal tear protein” to reflect a migration pattern that was different from albumin. Immunoelectrophoresis indicated that the protein was present in tears but absent in blood and other body fluids (Bonavida et al., 1969). It was renamed “specific tear prealbumin.” But tear lipocalin was unrelated to transthyretin and it migrated faster than albumin, and so it was rechristened “protein migrating faster than albumin” (Gachon et al., 1979). Later the primary sequence indicated the protein was a member of the lipocalin family. The sequence identity to family members was a maximum of 58% to von Ebner's gland protein of the rat, with only 27% identity to β lactoglobulin (Redl et al., 1992). For other human lipocalins the sequence identity is about 20–25% but specific residues such as tryptophan are highly conserved (Figure 2). The name was changed to “human tear lipocalin,” but was also referred to as von Ebner's gland protein as the same protein is secreted from lingual glands around circumvallate papillae (Delaire et al., 1992; Redl et al., 1992; Bläker et al., 1993; Lassagne et al., 1993). The glands had been named after the Austrian histologist, Victor von Ebner. Later, as one of the first lipocalins to be cloned, the gene for tear lipocalin was designated as “lipocalin-1,” while the name for the protein was retained as human tear lipocalin. This was a consensus decision made at Benzon Symposium #50 of the Lipocalin Protein Superfamily in 2003. The nomenclature is not strictly followed, so the protein is variously called tear lipocalin, lipocalin 1, and von Ebner's gland protein. Initial two-dimensional gel electrophoretic profiles revealed at least six published isoforms of tear lipocalin (Fullard and Kissner, 1991; Delaire et al., 1992; Glasgow, 1995). Later mass spectrometry demonstrated that these “isoforms” appear to be mainly truncated versions of the only protein predicted by a single known mRNA (Glasgow et al., 1998b). Native purified tear lipocalin contains all of these molecular species.

The nomenclature of the putative receptor for tear lipocalin is also relevant. Lipocalin-interacting membrane receptor was described as comprising of 647 amino acids, and the gene has 17 exons and at least five isoforms (Wojnar et al., 2001a). Several reports identified a nearly identical protein, limb development membrane protein-1, comprising of 649 amino acids. Most studies reported six sequential bases at the start of exon 3 that are missing in the original paper (Wojnar et al., 2001a). The isoform without the six bases is now considered as isoform 2, Q6UX01-2. The bases code for valine and aspartic acid in positions 53 and 54, respectively of the 649 amino acid receptor sequence. In almost all of the published variations of the sequence, the bases coding for valine and aspartic acid are present. Some other isoforms are reported as a result of theoretical and identified alternative splicing. The term limb development membrane protein-1 appears to be a misnomer. Initially, the genetic locus was linked to congenital limb malformations. Later it was shown that the malformation was due to the disruption of a long range sonic hedgehog enhancer located in an intron of the gene (Lettice et al., 2003). For simplicity, limb development membrane protein-1 will be considered synonymous with lipocalin-interacting membrane receptor. Here the term, lipocalin-interacting membrane receptor, will encompass the putative isoforms.

## Methods

### Gene Expression of Tear Lipocalin and Lipocalin-Interacting Membrane Receptor

To compare the mRNA expression profiles for tear lipocalin and lipocalin-interacting membrane receptor, the gene expression omnibus (GEO) repository was searched for human tissue profiles that contained either a variety of normal tissues and/or ocular surface tissues (Barrett et al., 2013). Each profile can include data derived from various sources such as expressed sequence tags (ESTs), microarrays, high throughput sequencing, nanostring methods, or reverse transcriptase polymerase chain reactions. Calculations are based on the original submitter-supplied expression measurements presented as “values” in the sample record. The submitters to the GEO repository are required to normalize the values of expression of each gene to the total number of expressed transcripts, as measured by their method. There is great diversity in the data values and ranges provided by GEO submitters. Each set may have different types of tissues and vary in types as well as number of transcripts that are tagged or identified. The presentation of data in the GEO repository can be variably formatted as relative values on linear or log base 10 scales or as log10 ratios representing the abundance of expressed transcripts. In each data set, the values are dimensionless. Submission of data to the GEO does not require the assessment of background noise in the data sets to ensure that a given threshold value represents true expression. Therefore, the expression of various tissues can be compared within a data set but are more difficult to compare between data sets. Specific data sets, GDS (GEO data set) (423,3834, 3113, 1085), were queried for expression values of the gene for lipocalin-interacting membrane receptor (Shyamsundar et al., 2005; Yanai et al., 2005; Dezs et al., 2008; She et al., 2009). In addition, GDS 2682 provides a comparison of gene expression in the conjunctiva and the cornea (Turner et al., 2007). In order to obtain the relative amounts of transcript expressed by lipocalin-interacting membrane receptor and tear lipocalin, the normalized data from a single data set were analyzed. However, in order to compare multiple data sets for the transcript expression of lipocalin-interacting membrane receptor rank means were used. This basic method avoids statistical misassumptions such as the presence of normal distributions. The relative order (rank) of the mean of the normalized values given for transcript expression of lipocalin-interacting membrane receptor was calculated for each type of tissue in a data set. A percentile rank was calculated for the expression of lipocalin-interacting membrane receptor for the various tissues of each data set. The percentile ranks of expression for each tissue type from the multiple data sets were then averaged to give a mean percentile rank to provide a broader view of the relative expression of lipocalin-interacting membrane receptor. Generally, a minimum of three samples were available for each tissue in any one data set. In one data set, the authors suggested a threshold value, below which it was considered noise (Yanai et al., 2005). Tissue types that had expression values below the threshold value of this data set were not considered for statistical analysis in other data sets without a threshold value. The analysis of such data is more useful if there is agreement across the data sets for a particular percentile rank of expression for a given tissue. The standard error of the mean of the mean percentile ranks is provided.

Since the lacrimal gland was not one of the tissues in the GEO data sets, data from the now discontinued Human UniGene (http://www.ncbi.nlm.nih.gov/unigene) libraries were included. The libraries were searched for tear lipocalin and lipocalin-interacting membrane receptor ESTs, and filtered with a minimum cut-off of 1000 transcripts. Expression levels were calculated by taking the sum of all ESTs in a given category divided by the sum of all transcripts.

### Identification of Lipocalin-Interacting Membrane Receptor in Ocular Surface Epithelium

**Figure 3:**
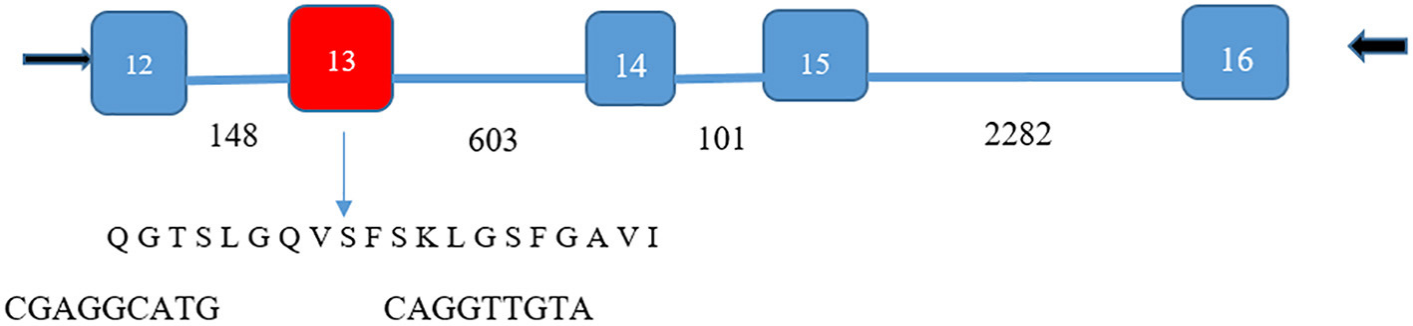
Isoform identification of lipocalin-interacting membrane receptor in ocular surface epithelium. PCR products of 499 and 559 base pairs spanned five exons (numbers shown in white) for the lipocalin-interacting membrane protein. Number of bases in the introns are shown by black numbers. The 60-base pair deletion in exon 13 (red) was verified by the sequencing of the 499 base pair product (DNA sequence cga ggc atg –cag gtt gta bridging the missing sequence). The missing 60-base pair sequence corresponds to amino acids missing from isoform 3, Q6UX01-3, an alternative splice variant (https://www.uniprot.org/uniprot/Q6UX01). This translates to the transmembrane helical segment of the protein sequence shown. The 559-base pair product contained the missing sequence.

Although quite useful as a guide, the GEO repository data for cornea and conjunctiva were not provided with threshold levels. Therefore, direct confirmation of transcript expression for lipocalin-interacting membrane receptor was undertaken. Reverse transcription polymerase chain reactions (PCR) were performed on cDNA from a human cornea epithelial library (ScienCell, Carlsbad, CA) as well as from discarded surgical samples of the cornea and conjunctiva in accordance with the Declaration of Helsinki. The study was approved by the UCLA Institutional Review Board for Human Subjects. Samples had generally been fixed in acidified ethanol for about 48 h. Cornea and conjunctival epithelium were microdissected, rinsed in RNAase free water, lysed, and homogenized in sterile microfuge tubes. RNA was extracted according to the instructions of the manufacturer (RNeasy Fibrous Tissue Kit (Qiagen). First strand cDNA was synthesized from the purified RNA using the iScript^™^ kit with RNase H+ reverse transcriptase (BioRad). Both first strand synthesis and PCR were done using a GeneAmp PCR system 2400 (Perkin Elmer). The strategy was to use lipocalin-interacting membrane receptor specific primers (forward 5′-GTGCTTGCTGGTGCTGACGG-3′ and reverse 5′-TCACTGGTGCTGGGTCTTCCTAGATG-3′) from exons that would have intervening introns (Figure 3). This ensured that genomic DNA was not misidentified as cDNA. In addition, some isoforms could be identified. The PCR parameters included 35 cycles of denaturation at 95°C, annealing at a step gradient including 60°C, 51°C, and 42°C for 20 s each, followed by extension at 72°C, with 1 min at each step. Products were immediately ligated in PCR 2.1TOPO vectors for 5 min and transformed in chemically-competent cells (Invitrogen). Selection of colonies and plasmid purification were performed as previously described (Gasymov et al., 1997). Sequencing was performed on an Applied Biosystems^®^ 3730 Capillary DNA analyzer (Life Technologies) using T7 and T3 promoter sas primers. Analysis of products from PCR and subcloned plasmid inserts was performed on 1.5–1.7% agarose gels stained with ethidium bromide.

## Results and Discussion

### Tissue Localization of Tear Lipocalin

**Figure 4:**
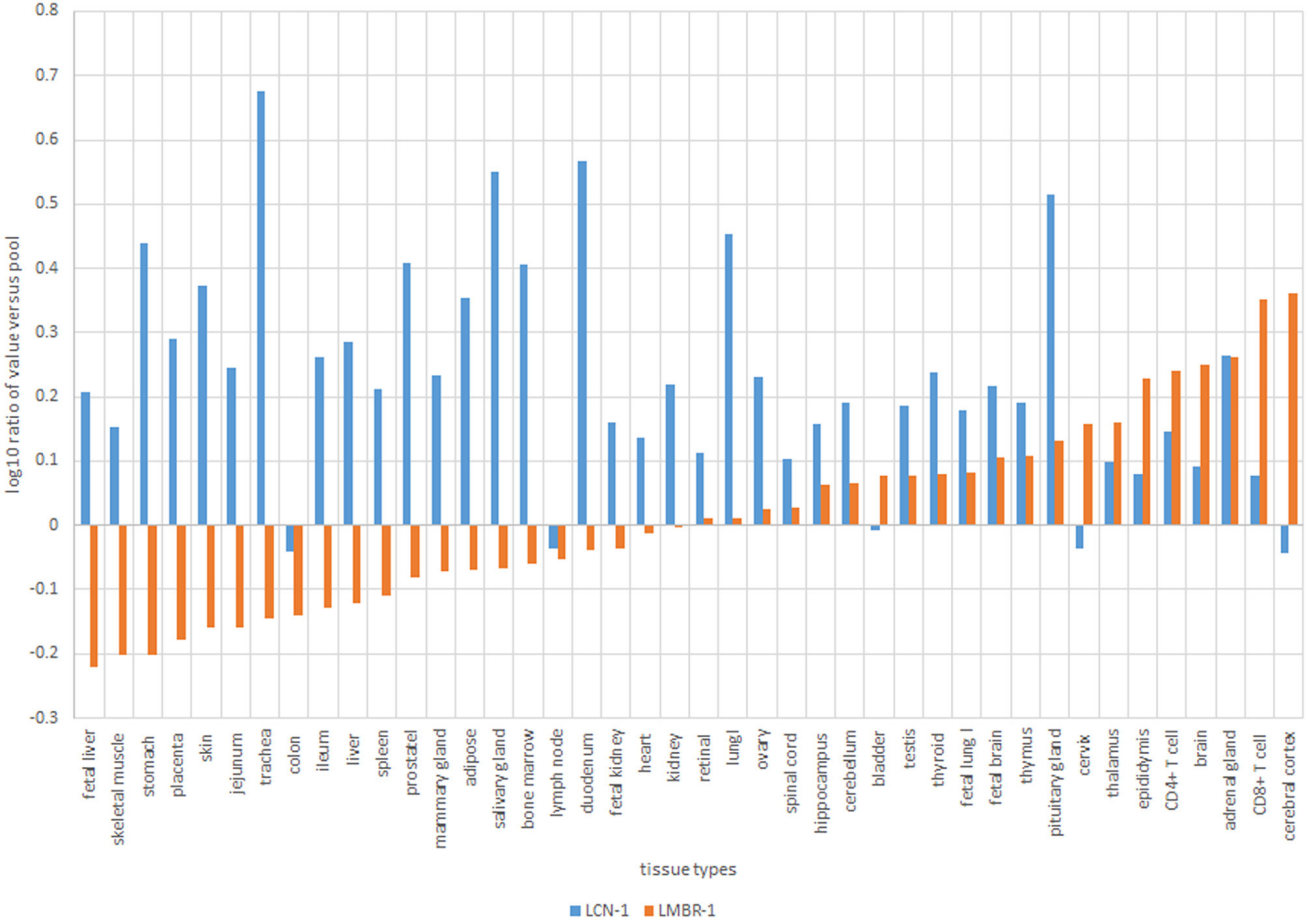
Normalized values of expression transcripts for tear lipocalin, blue (LCN-1) vs. lipocalin-interacting membrane receptor, orange (LMBR-1).

Initially, tear lipocalin was proffered to be tear specific. Immunofluorescence studies demonstrated tear lipocalins in acinar cells of main and accessory lacrimal glands (Glasgow, 1995; Ubels et al., 2012), but not in the cornea, meibomian glands, or conjunctiva (Inada, 1984). Tear lipocalin was identified by the group of Redl in the Western blots of saliva, sweat, and nasal mucus (Holzfeind et al., 1996; Wojnar et al., 2001a), as well as by immunofluorescence/immunohistochemical studies of the tracheobronchial tree, prostate, and pituitary (Holzfeind et al., 1995, 1996; Redl et al., 1998; Wojnar et al., 2002). Tear lipocalin could not be confirmed in eccrine glands of skin (Glasgow, 1995). This finding appears consonant with the expression value below threshold levels for noise (Table 1 and Figure 4). Immunoelectronmicroscopy studies of secretion of tear lipocalin in the lacrimal gland show that the protein appears to be packed in secretory granules often colocalizing with other proteins (Glasgow, 1995; Wojnar et al., 2002). The distribution was indicative of regulated secretion (Glasgow, 1995).

**Table 1:** Relative rank order of tissue expression of lipocalin-interacting membrane receptor from multiple GEO data sets.

**Tissue**	**Mean of means of percentile rank ± standard error**
Adrenal	6.4 ± 0.6
Testis	17.2 ± 10.4
Brain	21.2 ± 4.4
Thyroid	22.5 ± 10.1
Diaphragm	25.1 ± 2.6
Brain (fetal)	29.4 ± 1.9
Pituitary	30.0 ± 5.6
Lung	33.3 ± 10.0
Spinal cord	33.4 ± 11.6
Ovary	35.0 ± 8.5
Thymus (fetal)	37.5 ± 6.2
Breast	37.5 ± 12.5
Kidney	45.4 ± 6.2
Thymus	47.2 ± 20.3
Bladder	48.1 ± 13.1
Pancreas	50.0 ± 9.6
Uterus	51.9 ± 20.0
Blood (or lymphocytes)	53.1 ± 7.3
Prostate	55.0 ± 8.1
Pancreas	59.6 ± 9.6
Spleen	62.5 ± 19.7
Kidney (fetal)	62.8 ± 2.8
Noise threshold (Yanai et al., 2005)
Heart	64.7
Retina	66.9
Bone marrow	67.7
Liver	67.7
Salivary gland	71.4
Small intestine	72.2
Skin	78.8
Trachea	79.4
Tonsil	82.6
Placenta	84.7
Colon	89.1
Liver (fetal)	93.8
Vagina	94.4
Stomach	99.0
Skeletal Muscle	100.0

*The means of percentile ranks of normalized expression values for each tissue in every data set were averaged and ordered across the available data sets. Most abundant transcripts values have the lower ranks*.

### Tear Lipocalin Is a Multifunctional Protein

Gene-sharing is a common theme in evolution (Piatigorsky, 1998, 2007). Efficient conservation dictates that evolutionary selection pressure will favor proteins that are multifunctional. Tear lipocalin is paradigmatic for the multifunctional nature of proteins. The documented functions for this protein are shown in Table 2 and range from lipid-binding and transport, enzymatic activity, enzymatic inhibitor, and polymer formation. Virtually all the functions of the of tear lipocalin stem from interaction with other molecules. In most cases these are small hydrophobic lipids, proteins, or substrates such as DNA. Tear lipocalin is a promiscuous protein with a broad array of native ligands and the potential to bind an enormous suite of molecules. Table 3 shows many of the known ligands and interacting molecules of tear lipocalin. Direct comparison of the dissociation constants is often hindered by the methods used for calculation. Many have been calculated by displacement of a fluorescent ligand such as DAUDA, but some have been calculated directly. Table 3 shows that binding affinities are usually in the micromolar range. Tear lipocalin has an internal hydrophobic binding site with capacity for a C18 alkyl chain, although other hydrophobic groups can bind with less affinity (Abduragimov et al., 2000). The binding strength correlates with the length of the chain (Glasgow et al., 1995). The stoichiometric parameter (n) is close to 1 for the ligands of tear lipocalin. Data variation can be attributed mainly to preoccupied binding sites (Glasgow and Abduraguimov, 2018).

**Table 2:** Functions of tear lipocalin.

**Function**	**Interacting partner**	**References**
Scavenger of toxic compounds	Prostaglandins, ceramides, phospholipid, thioredoxin	Redl et al., 1999; Lechner et al., 2001; Wojnar et al., 2002; Gasymov et al., 2005; Glasgow and Abduragimov, 2018b
Transport of vitamins, nutrient	Vitamins A and E	Hong, 1986; Redl et al., 1992; Gasymov et al., 2002a; Glasgow et al., 2002a
Endonuclease activity	DNA (human/microbial)	Yusifov et al., 2000, 2008
Anti-microbial activity	Lipids, siderophores, lysozyme	Josephson and Wald, 1969; Kabara et al., 1972; Selsted and Martinez, 1982; Miller et al., 1988; Bibel et al., 1989; Fluckinger et al., 2004
Acceptor protein for phospholipid transport protein	Phospholipid transport protein, phospholipid	Glasgow and Abduragimov, 2021
Inhibitor of cysteine protease activity	Cysteine protease	Holzfeind et al., 1995; Van't Hof et al., 1997; Wojnar et al., 2001b
Viscosity (Confers non-newtonian sheer thinning behavior)	Polar lipids, lysozyme	Pandit et al., 1999; Tiffany and Nagyová, 2002; Gouveia and Tiffany, 2005
Drug delivery native tear lipocalin	Rifampin	Gasymov et al., 2004a; Staudinger et al., 2014
Drug delivery (Anti and duo-calins)	(e.g.,Vascular endothelial growth factor)	Eyer et al., 2012; Richter and Skerra, 2016

**Table 3:** Interacting molecules (ligands) native and non-native of tear lipocalin with binding constants.

**Ligand**	**Kd (μM) and/or special conditions**	**References**
Palmitic acid	8.3–13.5 tritiated palmitic acid apo/holo. 1.5–3.2 Ki	Glasgow et al., 1998a; Gasymov et al., 2011
Lauric acid spin label	2.4–8.3 apo/holo,	Gasymov et al., 1999a; Glasgow et al., 1999
Lauric acid	9.1 Ki apo	Gasymov et al., 1999a
Stearic acid	1.3 Ki apo	Gasymov et al., 1999a
Fatty alcohols C14-C18	NA	Glasgow et al., 1995, 1998b
16-(9-anthroyloxy)palmiticacid(16-AP)	0.8	Gasymov et al., 1999a
Cholesterol	15.9 Ki apo	Gasymov et al., 1999a
L-α-lysophosphatidylcholine	1.2–1.5 Ki and IC50	Gasymov et al., 1999a, 2005
2-(6-(7 nitrobenz-2-oxa,1,3-diazol-4-yl)amino)hexanoyl-1-hexadecanoyl-sn-glycero-3-phosphocholine (NBD C6-HPC)	0.15	Gasymov et al., 2005
Ceramide C6 NBD	0.08–0.32 (various methods)	Glasgow and Abduragimov, 2018b
Ceramide C12 NBD	0.1–1.23 (various methods	Glasgow and Abduragimov, 2018b
Retinol (Vitamin A)	0.13–0.19 apo pH7, pH3; 0.6 holo	Redl et al., 1992; Gasymov et al., 2002a; Breustedt et al., 2006
Retinoic acid	1.8	Breustedt et al., 2006
Retinal	0.39	Gasymov et al., 2002a
Vitamin E	>2.8 (displacement methods)	Glasgow et al., 2002b
Arachidonic acid	0.35 (IC50)	Lechner et al., 2001
7β-hydroxycholesterol	0.27 (IC50)	Lechner et al., 2001
8-isoprostane	0.94 (IC50)	Lechner et al., 2001
13-hydroxy-9,11-octadecadienoic acid	1.1 (IC 50	Lechner et al., 2001
4-Hydroxynonenal	16.8	Lechner et al., 2001
Lactoferrin and lysozyme	NA (shown by EPR	Gasymov et al., 1999b
Triacetylfusarinine C	0.5–1.5 (various methods)	Fluckinger et al., 2004
Rhodotorulic acid	~0.13(IC 50)	Fluckinger et al., 2004
Rifampin	128 (circular dichroism)	Gasymov et al., 2004a; Staudinger et al., 2014
Rifalazil	2.3 (centrifugal precipitation)	Staudinger et al., 2014
Rifabutin	22.3^*^ (gel filtration)	Gasymov et al., 2004a; Staudinger et al., 2014
Rifaximin	38.4^*^	Gasymov et al., 2004a; Staudinger et al., 2014
Rifamycin SV	63.8^*^	Gasymov et al., 2004a; Staudinger et al., 2014
Rifapentine	38.4^*^	Gasymov et al., 2004a; Staudinger et al., 2014
DAUDA(11-(((5-(dimeth-lyamino)−1-naphthalenyl)sulfonyl)amino)undecanoicacid)	1.0–2.8	Gasymov et al., 1999a; Lechner et al., 2001; Breustedt et al., 2006
ANS (8-anilino-1-naphthalenesulfonic acid)	0.5–10	Breustedt et al., 2006; Gasymov et al., 2008
1NPN,N-phenyl-1-naphthylamine	9.1	Gasymov et al., 2008
Fluorescein-labeled octadecyl ester	NA	Yeh et al., 2013
TNS, 6-(p-toluidino)-2-naphthalenesulfonic acid	7.6	Gasymov et al., 2008

*NA not available*.

* *Calculated from gel filtration relative to rifampin*.

### The Functions of Native Tear Lipocalin in Tears

The functions of a protein can be defined by the tasks that the ligand or the protein performs in native environments. Therefore, discovery of the native ligands provided important clues to functions. Tear lipocalin is highly concentrated in tears, ~60–100 μM, and is second only to lysozyme in concentration (Yeh et al., 2013). For tears the native ligands of tear lipocalin were extracted from the protein and include phospholipids, fatty acids, fatty alcohols, and cholesterol (Glasgow et al., 1995; Dean and Glasgow, 2012). Therefore, tear lipocalin obligatorily acts to solubilize, transport, and/or modulate these substances in tears. Most of the ligands have long alkyl chains and have negligible solubility in aqueous solutions. Transport of these ligands in tears to their targets allows the ligands to perform their functions. Conformational changes are triggered by local pH changes that promote ligand binding and release (Gasymov et al., 2007b). The following functions have been posited for tear lipocalin.

#### Antimicrobial Activity

Tear lipocalin facilitates antimicrobial activity by transport of antimicrobial lipids that would be otherwise be insoluble in tears. For example, lauric acid is a potent antimicrobial for *Propionibacterium acnes, Streptococcus group A, Nocardia* sp., *Micrococcus*, and *Candida* sp. (Kabara et al., 1972; Nakatsuji et al., 2009; Yang et al., 2009). Fatty alcohols have activity against herpes simplex virus (Sands et al., 1979). Tear lipocalin also has some bacteriostatic activity but it is not known if this is ligand-related or intrinsic to the protein (Selsted and Martinez, 1982). Tear lipocalin also interacts with lysozyme, which has a separate bacteriolytic function (Gasymov et al., 1999b). Interestingly, neither do the major lipids of the tear film, namely, wax and cholesterol esters, bind to lipocalin nor have they been shown to have significant antimicrobial activity (Glasgow and Abduragimov, 2018a). Tear lipocalin has antifungal activity. This action is related to binding fungal siderophores. The fungal siderophores compete with human lactoferrin for iron in tears. Iron is crucial for metabolic activities of microbes and host cells (Fluckinger et al., 2004). Antimicrobial activity might also be conferred by the ability of tear lipocalin to inhibit cysteine proteases, i.e., papain (Van't Hof et al., 1997; Wojnar et al., 2001b). Cysteine proteases cleave peptide bonds of proteins at a thiol group adjacent to a basic amino acid, commonly histidine. Microbial cysteine proteases cleave inactive precursors of microbial proteins to create active forms. Inhibition of cysteine protease prevents cleavage and may disable microbial functions. The prototypical cysteine protease inhibitor is cystatin, which shares some common sequences with tear lipocalin. The N terminus of tear lipocalin confers the inhibition of the protease activity, perhaps by blocking substrate access. At high concentrations of papain, the inhibition by tear lipocalin was vitiated due to cleavage of the N terminus of tear lipocalin. Some forms of cystatin have greater inhibitory activity than tear lipocalin (Wojnar et al., 2001b). Inhibition of cysteine proteases by tear lipocalin has not yet been related to the inhibition of any specific microbes. The relative importance of cysteine protease inhibition of tear lipocalin remains uncertain.

Tear lipocalin may have an antimicrobial role in the mouth. However, it has also been posited that tear lipocalin carries small molecules to receptors for taste, but experiments testing the binding of tastants have not been successful (Schmale et al., 1993).

#### Transport of Vitamins E and A

Tear lipocalin has been shown to bind retinol and vitamin E. Vitamin E has been extracted from purified fractions of tear lipocalin from tears. About half of the Vitamin E content is bound to protein and 86% of the protein bound fraction is complexed to tear lipocalin (Glasgow et al., 2002b). Vitamin E is a potent antioxidant and potentially useful to the exposed lipid layer of the tear film as well as to ocular surface epithelium.

Retinol, retinal, and retinoic acid have been shown to bind lipocalin but have not been extracted successfully from purified lipocalin in tears (Redl et al., 1992; Gasymov et al., 2002a). Retinol binding protein has a higher affinity and specificity for retinol than lipocalin. It remains to be seen what the role for tear lipocalin is in the transport of retinol from the tear film to the ocular surface epithelium.

#### Scavenger for Lipid Peroxidation Products

Tear lipocalin was studied in a teratocarcinoma cell line and found to have increased expression when treated with ferrous sulfate or hydrogen peroxide (Lechner et al., 2001). In the same study, centrifugal concentration followed by anion exchange chromatography was used to enrich fractions of tear lipocalin from the cell culture. An enzyme immunoassay of chloroform extraction products of fractions with tear lipocalin was positive for F2-isoprostanes. *In vitro* DAUDA displacement assays confirmed that arachidonic acid and several lipid peroxidation products including 7β hydroxycholesterol, 8-isoprostane, and 13-hydroxy-9, 11-octadecadienoic acid were bound to tear lipocalin. The conclusion was that tear lipocalin has an essential function in scavenging harmful lipid peroxidation compounds. Further, this scavenger function was related to the interaction of tear lipocalin with thioredoxin. This interaction was discovered by the phage display of a cDNA library of prostate tissue. Thioredoxin appears to promote oxidation of the conserved disulfide bond in tear lipocalin. The proposed function is that the promotion of disulfide oxidation results in an increased affinity of tear lipocalin for a number of toxic ligands. This would have an impact on circumstances where disulfide reduction is favored, such as anaerobic conditions (Redl et al., 1999). However, the disulfide bond of tear lipocalin is normally intact in tears and proof of this mechanism has not been substantiated (Glasgow et al., 1998b).

#### Scavenger for Lipids From the Surface of the Cornea and in Tears

Tear lipocalin has been shown to remove native lipids from a variety of surfaces including the ocular surface (Glasgow et al., 1999; Gasymov et al., 2005; Yeh et al., 2013). Many meibomian lipids are hydrophobic due to long alkyl chains, and often these lipids are insoluble in aqueous solutions. Lipid contamination of the corneal surface, either because of loss of mucin or contamination of a mucinous surface, lowers the surface tension and renders the cornea unwettable (Sharma, 1993). This situation is possible whenever the tear film thins, such as in dry eye disease. The types of lipids shown to be removed from the ocular surface include fatty acids and phospholipids. Tear lipocalin also binds avidly to ceramides (Glasgow and Abduragimov, 2018b). Ceramides may destabilize the tear film. Ceramides have been shown to increase hysteresis in Langmuir trough experiments and can induce eventual collapse of the lipid film (Arciniega et al., 2013). Ceramides comprise about 7% of the total lipids in chalazia (Nicolaides et al., 1988). Elevation of ceramides has been noted in moderate dry eye disease (Lam et al., 2011). The evidence suggests that a principal function of tear lipocalin is to solubilize, sequester, and shuttle potentially destabilizing lipids from the ocular surface to the nasal lacrimal duct.

#### Acceptor for Phospholipid Transfer Protein and Modulator of Phospholipids

Recently, tear lipocalin has been shown to accept phospholipids from micelles in tears in concert with phospholipid transfer protein (Glasgow and Abduragimov, 2021). Since micelles have not been found in tears one cannot be sure whether micelles were present in the first place. However, the lipid composition in tears has been modeled to be conducive to forming both normal phase and inverse micelles (Wizert et al., 2014). The concentration of these lipids in tears exceeds the critical micellar concentration. Micelles scatter light. The intensity of the light scatter is a function of the square of the difference of the index of refraction of micelles from aqueous solution (Rayleigh, 1899). Elimination of micelles would reduce scattered light and maintain clarity of tears, a critical requirement for vision. Phospholipids have been shown to exist at the surface of the lipid layer of the tear film (Glasgow, 2020). Despite their surface activity, phospholipids are probably not present in enough concentrations to form a monolayer on tears (Glasgow, 2021). Evidence for the amount of phospholipid at the tear surface is based on the absorption from native phospholipid in stimulated tears compared with a known monolayer of phospholipid (Glasgow, 2020). But even small amounts of phospholipids will aid in the spreading of the main lipid components, such as cholesterol and wax esters, of the tear film (Rantamäki and Holopainen, 2017). A greater amount of phospholipid could theoretically displace gel-forming lipids from the tear film. Modulation of phospholipid concentrations by tear lipocalin could provide a more stable tear film.

#### Modulation of Viscosity in Tears Through Heteroprotein Polymer Formation

Tear lipocalin may have a role in modulating the viscosity of the tear film, although the data are confusing. The tear film behaves as a non-Newtonian fluid, meaning that the viscosity of tears is dependent on the sheer stress. Viscosity in Newtonian fluids, such as water, is independent of sheer stress. The source of sheer stress for tears is the blinking of the eyelids over the ocular surface. Tears are sheer thinning such that the viscosity decreases with increased blink speed (Gouveia and Tiffany, 2005). Sheer thinning with lower viscosity may vitiate damage to the ocular epithelium during blinking. The usual basis for sheer thinning is polymer separation often from the reduction in hydrogen bond interactions. One would posit that polymers exist in tears that are separated with blinking. The observation made by the group of Tiffany was that the removal of lipids from tears (most lipids are bound to tear lipocalin) results in Newtonian or sheer independent behavior, suggesting that the presence of lipids contributes to polymer formation. However, recombinant tear lipocalin in the work of Tiffany, presumably apo-tear lipocalin, showed non-Newtonian sheer thinning behavior. This seeming contradiction may be explained by the observation that recombinant tear lipocalin expressed in *E. Coli* has bound lipids (Gasymov et al., 2007c; Tsukamoto et al., 2009). But what is unexplained is that holo-tear lipocalin (with tear lipids added back to recombinant tear lipocalin) also showed Newtonian behavior (Gouveia and Tiffany, 2005). This appears contradictory to data that show recombinant and holo-tear lipocalin are monomeric, whereas aggregation occurs with delipidation (Gasymov et al., 2007c). The molecular basis of the polymer interaction in tears remains unclear. Perhaps, the relatively weak electrostatic interaction documented between tear lipocalin, lysozyme, and lactoferrin may have some influence (Gasymov et al., 1999b).

#### Surface Activity in Tears and Reduction of Evaporation

Tear lipocalin, like many proteins including some tear proteins, has been shown to unfold at an aqueous–air interface (Glasgow et al., 1999; Tragoulias et al., 2005; Mudgil and Millar, 2008). Further, tear lipocalin was shown to insert into meibomian film layer (Miano et al., 2005). The rate of penetration seems to differ between bovine and human meibomian layers and the reason for the difference is not entirely clear (Mudgil and Millar, 2008). Compared to other tear proteins, lipocalin was able to penetrate even at 30 mN/m of surface pressure, the highest surface pressure attained by whole tears. One possible mechanism that has been proposed for penetration of the surface by tear lipocalin is a proton conduction gradient, which forms at interfacial planes between lipids and aqueous (Prats et al., 1987). Phospholipids have been shown to produce a pH gradient when present at an aqueous interface. Phospholipids are present at the surface of tears (Gabriel et al., 1991; Glasgow, 2020). Acidic pH induces structural changes in proteins, and in this case it particularly facilitates loop motion of tear lipocalin. Changes in loop conformation result in low affinity for lipids, for example, some lipids may be offloaded at the surface (Gasymov et al., 1998, 2010a,b). Lipids, particularly long chain fatty alcohols, are known to reduce evaporation (La Mer and Healy, 1965; Saggaï and Bachi, 2018). The data for the effect of tear lipids and proteins on evaporation are somewhat disparate and controversial (Borchman et al., 2009; Herok et al., 2009). Tear lipocalin was not specifically tested in these studies. While proteins appear to contribute marginally to reducing evaporation in tears, surface active lipids should have a significant effect.

#### Endonuclease Activity

Tear lipocalin has been shown to be the major endonuclease in tears, accounting for about 75% of total endonuclease activity (Yusifov et al., 2000, 2008). Tear lipocalin acts as a Mg^+2^-dependent nonspecific endonuclease. Activity has been related to a conserved protein sequence motif, LEDFXR, which is shared by *Serratia marcescens*. The LEDFXR motif is found in other lipocalins with similar activity (e.g., bovine β-lactoglobulin). Those lipocalins lacking the motif show no activity (e.g., retinol-binding protein). Glutamine at position 128 in tear lipocalin is critical for endonuclease activity. The functional relevance of endonuclease activity may be to degrade human DNA from exfoliated epithelial cells of the ocular surface. In addition, degradation of microbial DNA by tear lipocalin would serve as a useful function for destroying potentially infectious DNA of viruses. Such activity may work in concert with lysozyme in tears that binds viral DNA (Lin et al., 2008).

### Drug Delivery

Because of promiscuity for ligands, tear lipocalin and mutant proteins derived from tear lipocalin have been used as drug binders and transporters. Tear lipocalin binds avidly to several members of the rifamycin family (Table 3). This raises the possible application of treatment for tuberculosis. Not only do these drugs bind lipocalin, but some rifamycins that are susceptible to oxidative degradation are protected when bound to tear lipocalin (Gasymov et al., 2004a; Staudinger et al., 2014).

Mutations of the loops at the open end of the cavity of lipocalins may result in specific changes in binding. Using combinatorial libraries the Skerra's group has made mutants of several lipocalins for specific drug-like action. For example, a mutant of tear lipocalin can act to bind vascular endothelial growth factor, and it is in the pipeline as a possible therapy (Hohlbaum and Skerra, 2007).

### Biomarkers

Tear lipocalin has been touted as a biomarker for several diseases including dry eye disease (Zhou et al., 2009; Karnati et al., 2013; Yeh et al., 2013), breast cancer (Yang et al., 2019), chronic obstructive pulmonary disease (Jessie et al., 2010; Nicholas et al., 2010; Wang et al., 2014), diabetic retinopathy (Csosz et al., 2012; Guzman et al., 2020), glaucoma and pseudoexfoliation syndrome (Pieragostino et al., 2012, 2013), Alzheimer's disease (Kall et al., 2016), and keratoconus (Pannebaker et al., 2010; Acera et al., 2011). Most of these studies are based on proteomic correlations or expression profiles with elevated or depressed lipocalin levels. In the case of dry eye disease, one would expect that protein secretion of tear lipocalin as well as lysozyme and lactoferrin would be impacted together by the destruction of lacrimal gland acini. These proteins are packaged and secreted together in the lacrimal gland (Glasgow, 1995). Further testing of large population groups is warranted to validate the use of tear lipocalin as a biomarker in these settings.

## Structure Function Relationships of Tear Lipocalin

The solution structure (by site directed tryptophan fluorescence) and later the crystal structure (by X-ray crystallography) of tear lipocalin have been reported (Gasymov et al., 2001; Breustedt et al., 2005). There is a remarkable concordance between the published structures (Figure 1). The solution structure was obtained by deducing the molecular environment of sequentially substituted tryptophans to yield an accurate map of secondary structural elements throughout the entire protein. The map was transposed to align secondary elements and the sequence compared with a close relative in the lipocalin family, whose structure was known. Computer-calculated energy minimization for favored conformations resulted in the most probable 3D structure. The solution structure is especially informative in defining loop regions that were not sufficiently resolved by crystallography (Figure 1). The functional features of various structural elements (Table 4) were defined by studies in solution, but residue interactions were confirmed by their proximity with crystallography. Unlike other lipocalins, tear lipocalin has the capacity to bind a broad array of ligands (Table 3). The cavity is 10 Å in diameter and 15 Å deep. Binding affinity increases for alkyl chains up to 18 carbons in length. The loops are critical to both the affinity and specificity of ligand binding. In some lipocalins, the length of the overhanging hairpin loop EF constrains the ability of the ligand for conformational selection to enter the cavity. Retinol binding protein is relatively specific for retinol due to the constraints of a long EF loop. The EF loop is short in tear lipocalin, allowing greater-sized ligands access to the cavity. The motion of the AB, GH, and CD loops, as detected using fluorescence quenching techniques, have been shown to be critical for pH-driven ligand binding (Gasymov et al., 2004b, 2010b). A protonated residue appears to be the trigger for loop motion at low pH. This leads to a low affinity conformation state and favors the release of the ligand.

**Table 4:** Structure-function of critical motifs of tear lipocalin.

**Structural Motif**	**Amino Acid, Strand**	**Function**	**References**
Hydrophobic patches	• W17/F99 **A** & **G** internal • I98 **G** external	Stabilizes ligand binding through rigidity, conserved	Gasymov et al., 1998, 2002a,b
Disulfide bond	• C79 & C171 • **D** strand & C terminus	Induces protein rigidity, aromatic asymmetry; modulates conformational selection, conserved	Glasgow et al., 1998b
N terminus segment	L4-S7 & Q12-G16 **A**	Cysteine protease inhibition	Van't Hof et al., 1997; Wojnar et al., 2001b
Titratable trigger residues	R27 (**AB**)	Protonation triggers loop motion regulates pH dependent ligand binding	Breustedt et al., 2009; Gasymov et al., 2010b. PDB files: 1XKI, EYC
Calyx entry loops	**AB** and **GH**	Critical for conformational selection of ligands, pH modulated	Gasymov et al., 2009, 2010b
Calyx entry loop	**CD**	Conformational selection, constrained by disulfide	Gasymov et al., 2004b
Calyx entry loop	**EF** (hairpin)	Ligand specificity of cavity, short length add promiscuity; large range of motion	Gasymov et al., 2001, 2004b
Closed end loop	**FG**	Possible receptor recognition site, highly conserved	Gasymov et al., 2000, 2001, 2009
Cation-π	• K108-F28 **H** &**AB** • R118-W17 **H** &**A**	Stabilization of holo-conformation, conserved	Gasymov et al., 2012a
Trigonal cluster	K114 **H**, H84 **F**, E34 **AB**	External binding site for charged residues	Gasymov et al., 2007a, 2008
Mg^+2^ H_2_0 cluster	E128 ***α***	Endonuclease activity, divalent cation dependent, conserved	Yusifov et al., 2000
α- helix strand	F130 **α**- V113&I115 **H**	Interaction modulates strand motion for long range residue interactions	Gasymov et al., 2013

*Amino acids are designated with single letter and numbered residue position. β strands, loops, and α-helices are designated in bold font with single letters, two letters or **α**, respectively*.

Recently, cation-π interactions between positively charged residues and the negative charge cloud in the benzyl ring of aromatic amino acids were found (Gasymov et al., 2012a). Cation-π interactions are highly conserved among all lipocalins. The interactions stabilize the binding cavity, particularly in the holo state. Tear lipocalin contains motifs composed of one or more amino acids that are in proximity in the three-dimensional structure, but separated by long distances along the polypeptide chain of the protein. Substituting a residue to change either the charge, hydrophobicity, or size may greatly alter the binding affinity and function of tear lipocalin without significantly altering the secondary structure. For example, substituting alanine with tryptophan at residues 51, 66, and 86 in tear lipocalin reduced binding affinity for ligands by 3–4-fold (Gasymov et al., 2001). Substitution of E128 with tryptophan in tear lipocalin, reduced endonuclease (DNA nicking) activity by 80% (Yusifov et al., 2000).

Solution structure studies with ligands, both native and non-native, have been used to study quenching of single tryptophan mutants in tear lipocalin (Gasymov et al., 2009). Here the advantages of solution structure over crystal structure emerge. Dynamic loop structure is easily resolvable in the solution structure studies. One can probe the proximity to ligands to many amino acid residues of the protein in real time to provide a distribution of ligand binding sites. The distribution is reflective of multiple conformational states. Crystallography creates a model of a static structure. Only one conformation is usually sampled. Two crystal structures are available for tear lipocalin (Breustedt et al., 2005, 2009). The solution structure of tear lipocalin shows that residues on loops AB and GH as well as strands G and H are predominant interaction sites for both native and non-native ligands. Ligands move about the tear lipocalin cavity and loops; their positions are not static. Further, loops AB and GH at the open end of the cavity form the key portal for conformational selection by ligands in tear lipocalin (Gasymov et al., 2009). Based on ligand quenching of fluorescent residues, both static and dynamic, the major cavity-binding sites reside on the G and H strands in the cavity. The native ligand reports indirect quenching of mutant sites by amino acids in areas other than the binding site. For example, the FG loop moves to a more solvent-exposed conformation. An exposed conformation with ligand binding implicates the loop in potential receptor interactions (Gasymov et al., 2009). This is a plausible explanation for the highly conserved nature of this loop throughout the lipocalin family. Distance measurements between residues can be probed to the angstrom scale with variations of site-directed tryptophan fluorescence to detect subtle intramolecular changes such as rotamer conformations (Gasymov et al., 2012b, 2015).

The importance of portal loops was recognized by the group of Skerra (Schlehuber and Skerra, 2005; Gebauer and Skerra, 2012). Combinatorial methods facilitated variable mutations at positions of the loops in several lipocalin structures. The mutations can alter the conformations the lipocalins present to ligands. Anticalins, duocalins, etc. have been constructed for novel ligands with strong affinity. The strategy has worked well not only to create laboratory reagents for assays, but also for screening mutant proteins as potential treatments to target specific molecules involved in pathways in diseases. For example, tear lipocalin mutants with altered residues in the loops successfully target vascular endothelial growth factor, potentially useful in forms of macular degeneration and neoplasms. One caveat is that the loops of the lipocalin are exposed. The exposed nature of an altered sequence may pose a risk of increased antigenicity.

## The Putative Receptor for Tear Lipocalin, Lipocalin- Interacting Membrane Receptor

Receptors for the lipocalin family have been difficult to identify as is exemplified in the story of the receptor for retinol binding protein (Kawaguchi et al., 2007). A putative membrane receptor for tear lipocalin has important implications for potential functions. Lipocalin-interacting membrane receptor was discovered by biopanning a bacteriophage cDNA expression library from the human pituitary gland with purified tear lipocalin (Wojnar et al., 2001a). The genomic structure with 17 exons was also reported by the group of Redl (Wojnar et al., 2001a). The N terminus of the 487-amino acid protein interacted with purified tear lipocalin and was localized to the plasma membrane of NT2 cells derived from pluripotent human embryonal carcinoma (Wojnar et al., 2001a,b). The protein was modeled with nine transmembrane helical domains. The detailed structure is not known. Downregulation of lipocalin-interacting membrane receptor resulted in inhibition of cellular internalization (NT2 cells) of tear lipocalin (Wojnar et al., 2003). Therefore, lipocalin-interacting membrane receptor was considered an endocytic receptor. However, similar results were obtained for internalization of bovine β lactoglobulin by lipocalin-interacting membrane receptor (Fluckinger et al., 2008). Curiously, forced expression of the lipocalin-interacting membrane receptor in COS-1 cells facilitated the binding of uteroglobin as well (Zhang et al., 2006). In contrast, surface plasmon resonance experiments with expressed lipocalin-interacting membrane receptor showed an interaction with tear lipocalin but not with β lactoglobulin or uteroglobin (Hesselink and Findlay, 2013). Recently, the function of lipocalin-interacting membrane receptor as an endocytic receptor for tear lipocalin has been challenged. An analogous protein to lipocalin-interacting membrane receptor in *Drosophila*, coined Lilipod, was shown to function in self-renewal of ovarian germ-line stem cells through enhanced signaling of bone morphogenetic protein (Dolezal et al., 2015). The functions described for these closely related proteins are not necessarily mutually exclusive. Comparison of various tissues for expression of both tear lipocalin and lipocalin-interacting membrane receptor may provide insight into their potential interactions.

### Repository Data for Expression of Lipocalin-Interacting Membrane Receptor and Tear Lipocalin

**Figure 5:**
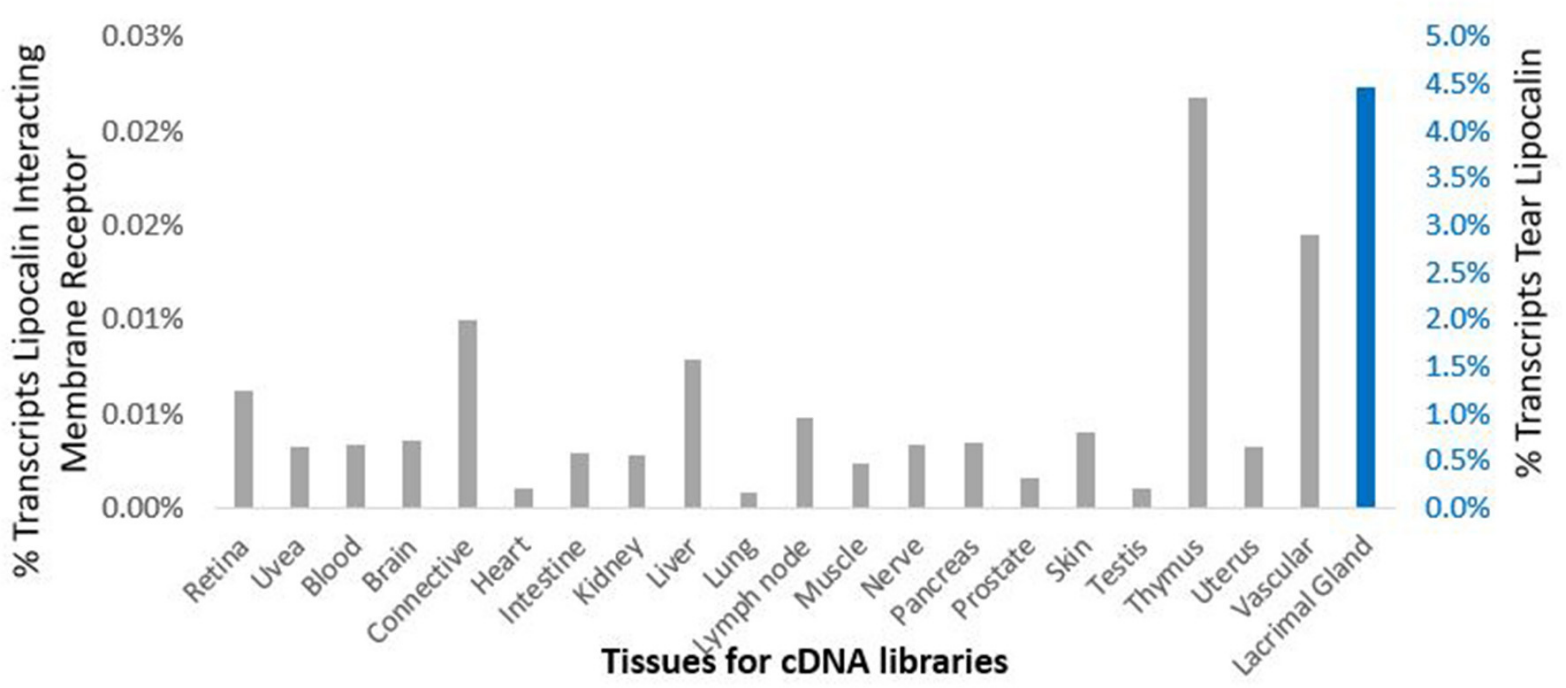
Comparison of cDNA libraries from multiple human tissues for expression of lipocalin-interacting membrane receptor (gray) and tear lipocalin (red). The y-axis shows the percent of the total transcripts. The secondary *y*-axis, right is scaled differently for tear lipocalin because of the dramatic abundance of this transcript in the lacrimal gland library such that none of the other values appear in the bar graphs. The next most abundant value of percent total transcripts for tear lipocalin was in the testis 0.003%. Data was provided in the Unigene data base.

**Figure 6:**
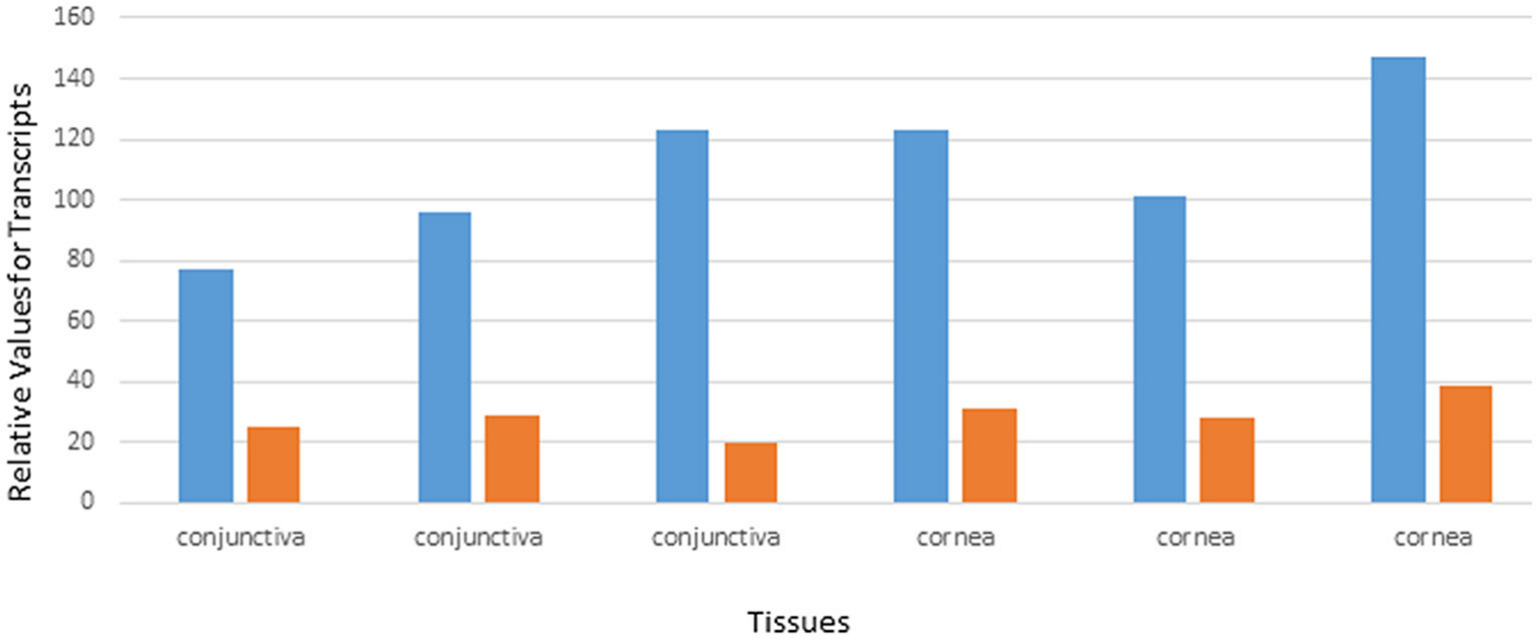
Relative values of expression transcripts for tear lipocalin (orange) vs. lipocalin-interacting membrane receptor (blue) for cornea and conjunctiva samples. The data are adapted from GDS profile 2682 in the gene expression omnibus.

Data from the GEO repository is useful to roughly assess the relative abundance of lipocalin-interacting membrane receptor transcripts in normal human tissues. An overall perspective from multiple data sets is provided in Table 1. The adrenal gland is at the top percentile ranking and the low standard error of the mean indicates that the expression of lipocalin-interacting membrane receptor was consistent across the data sets. The findings from brain tissue were similar to those of the adrenal gland. On the other hand, skeletal muscle and heart showed consistently lower percentile ranks of expression. In one study, a threshold below which values were considered noise and not associated with transcripts occurred for heart, liver, and skeletal muscle, as indicated in Table 1 (Yanai et al., 2005). The adrenal gland and brain are not known either to express tear lipocalin or have access to these organs through the blood stream. The expression data question the notion whether lipocalin-interacting membrane receptor is a specific receptor for tear lipocalin. An attempt to specifically correlate the relative expression of lipocalin-interacting membrane receptor with tear lipocalin was possible in two large data sets. Figure 4 shows the data for one such data set as log 10 values (She et al., 2009). Perhaps serendipitously, the log 10 format includes values from the heart at log values corresponding to the level of noise in Table 1. Adrenal gland and brain showed the highest expression of lipocalin-interacting membrane receptor. Furthermore the expression profiles of tear lipocalin (Figure 4) match the immunohistochemically identified expression of protein in tissues, such as trachea, prostate, and pituitary gland. However, for most tissues, the levels of expression were disparate for the two proteins. The lack of correlative expression was evident in another data set of 96 tissues (Dezs et al., 2008). Of course, expression profiles do not necessarily have to be similar to have a link in function (Yanai et al., 2006). Lacrimal gland tissue was not used in the studies retrieved from the GEO repository. However, the Unigene database included lacrimal gland tissue. The percentage of ESTs of tear lipocalin in the lacrimal gland library was three orders of magnitude more than the percentage of those for lipocalin-interacting membrane receptor in other tissue libraries (Figure 5). The percentages of tear lipocalin ESTs in libraries other than the lacrimal gland appear negligible. Cornea and conjunctiva tissues were not included in the aforementioned studies. However, these ocular surface tissues were present in one data set (Figure 6) in the GEO repository (Turner et al., 2007). The value of expression transcripts of lipocalin-interacting membrane receptor is greater than tear lipocalin in both conjunctiva and cornea. However, actual levels of expression relative to potential noise are not certain. The functional implications of the expression of lipocalin-interacting membrane receptor in ocular surface cells that are bathed in tears containing high concentrations of its putative ligand, tear lipocalin, calls for confirmation.

### Ocular Surface Expression of Lipocalin-Interacting Membrane Receptor

**Figure 7:**
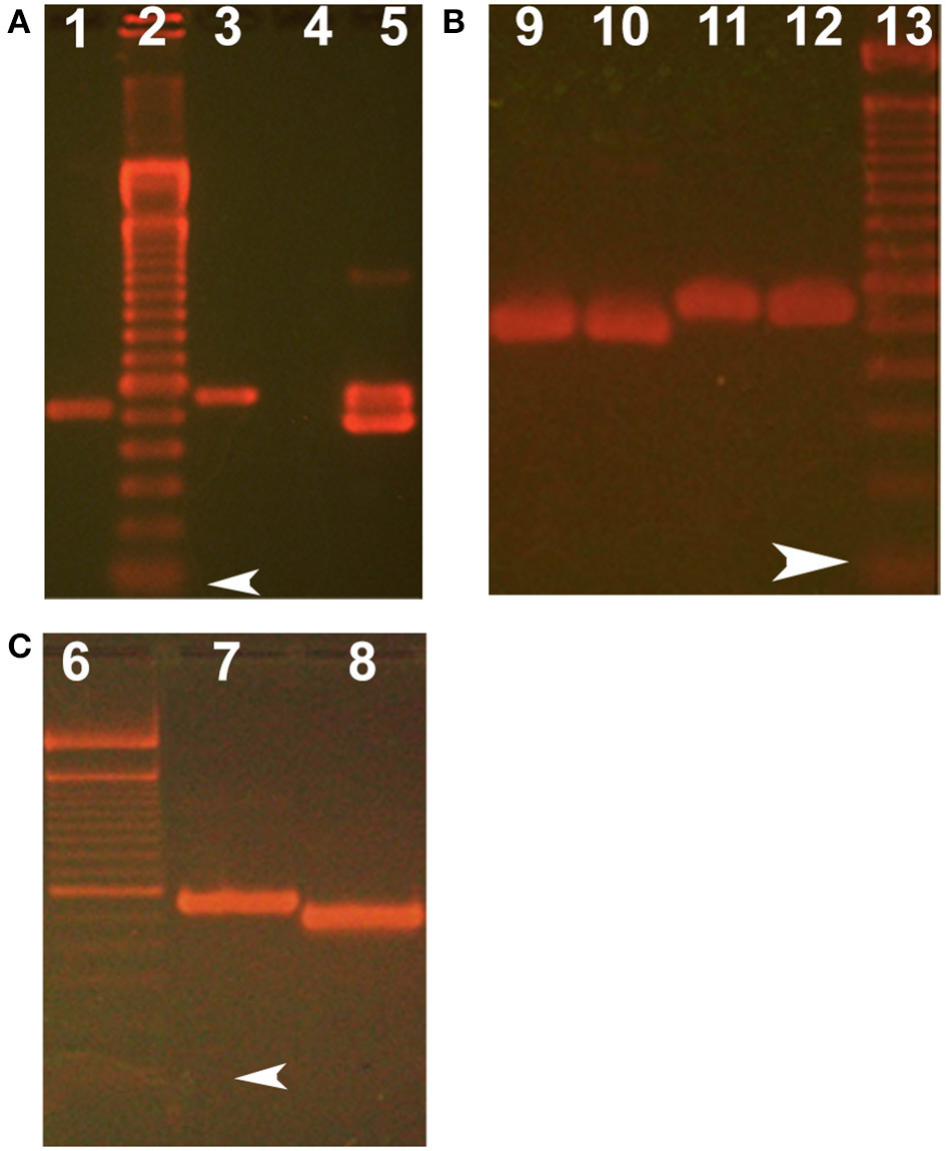
Ethidium bromide stained agarose gels show size of products from PCR and subcloning lipocalin-interacting membrane receptor. **(A)** Shows RT-PCR products from corneal epithelium. **(B)** Shows plasmid inserts from single colonies from subcloning of RT- PCR of conjunctiva. **(C)** Shows plasmid inserts from single colonies from subcloning the PCR products of the corneal epithelial library. Lanes 2, 6, and 13 show 100 base pair molecular weight markers, arrowheads show the first marker at 100 base pairs. Target products lane 1 keratin 14 (control for cornea) using forward primer 5′-AGCCGCATTCTGAACGAGAT-3′ and reverse primer 5′-TCGTGCACATCCATGACCTT-3′, expected product size 529 bases; lanes 3, 5–10 show lipocalin-interacting membrane receptor using forward primer 5′-GTGCTTGCTGGTGCTGACGG-3′ and reverse primer 5′-TCACTGGTGCTGGGTCTTCCTAGATG-3′). Lane 3 uses plasmid with cloned lipocalin receptor as a positive control. Lane 4 is negative control (absence of template). The two sized products (e.g., Lane 5) were shown by sequencing to represent isoform, one which lacks a 60 base pair sequence corresponding to amino acids missing from isoform 3, Q6UX01-3, an alternative splice variant (as shown in Figure 3) (https://www.uniprot.org/uniprot/Q6UX01).

The presence of lipocalin-interacting membrane receptor transcripts was queried in cultured human corneal epithelium cDNA (hCEPIC-ScienCell). The strategy was to amplify segments of the cDNA that spanned introns to exclude genomic contamination. The identification of a known alternative splicing isoform and the presence of large introns encompassed by the primer sets exclude the possibility of contamination by genomic DNA (Figure 3). The amplification strategy included two possible isoforms of lipocalin-interacting membrane receptor. The PCR products of individual colonies are shown in Figure 7. Two distinct sizes of 499 and 559 base pairs matched lipocalin-interacting membrane isoforms. and this was confirmed by sequence data (Figure 3). The agarose gel of PCR products from cDNA extracted from corneal epithelium is shown in Figure 7. Multiple products were similar in size to those seen from the corneal epithelial cell cDNA library as well as the conjunctiva. The expression of lipocalin-interacting membrane receptor at the ocular surface supports a possible receptor interaction with tear lipocalin. One possibility is that tear lipocalin functions to deliver vitamins or other molecules to the avascular central cornea epithelium through the receptor. Lacking here is immunohistochemical evidence of protein translation of lipocalin-interacting membrane receptor at the ocular surface. This was attempted but commercial antibodies as well as those obtained from Professor Redl did not show reactivity in tissues fixed in methanol, acidified ethanol, or formalin. Lipocalin-interacting membrane receptor, as well as tear lipocalin may be multifunctional proteins, which might explain some disparities. More investigation is needed to clarify the functions of this membrane protein, particularly in regard to its structure, distribution, and interactions with tear lipocalin.

## Data Availability Statement

The datasets for this study can be found in the Gene Expression Omnibus, https://www.ncbi.nlm.nih.gov/geo/, Protein Data Bank http://www.wwpdb.org/, The sequence data have been deposited in Gene Bank, accession numbers, MW841072 and MW841073.

## Ethics Statement

The studies involving human participants were reviewed and approved by UCLA Institution Review Board. Written informed consent for participation was not required for this study in accordance with the national legislation and the institutional requirements.

## Author Contributions

The author confirms being the main contributor of this work and has approved it for publication.

## Conflict of Interest

The author declares that the research was conducted in the absence of any commercial or financial relationships that could be construed as a potential conflict of interest.

## Publisher's Note

All claims expressed in this article are solely those of the authors and do not necessarily represent those of their affiliated organizations, or those of the publisher, the editors and the reviewers. Any product that may be evaluated in this article, or claim that may be made by its manufacturer, is not guaranteed or endorsed by the publisher.
